# Association of fall rate and functional status by *APOE* genotype in cancer survivors after exercise intervention

**DOI:** 10.18632/oncotarget.28310

**Published:** 2022-11-17

**Authors:** Gwendolyn J. McGinnis, Sarah Holden, Betty Yu, Charlton Ransom, Carolyn Guidarelli, Brian De, Kevin Diao, David Boyce, Charles R. Thomas, Kerri Winters-Stone, Jacob Raber

**Affiliations:** ^1^Department of Radiation Oncology, The University of Texas MD Anderson Cancer Center, Houston, TX 77030, USA; ^2^Department of Behavioral Neuroscience, Oregon Health and Science University, Portland, OR 97239, USA; ^3^School of Nursing, Oregon Health and Science University, Portland, OR 97239, USA; ^4^Department of Radiation Medicine, Oregon Health and Science University, Portland, OR 97239, USA; ^5^Department of Radiation Oncology, Dartmouth-Hitchcock’s Dartmouth Cancer Center, Lebanon, NH 03756, USA; ^6^Knight Cancer Institute, Oregon Health and Science University, Portland, OR 97239, USA; ^7^Department of Neurology and Division of Neuroscience, ONPRC, Oregon Health and Science University, Portland, OR 97239, USA; ^*^Joint last authors

**Keywords:** apoE, breast cancer, exercise intervention, fall rate, functional status

## Abstract

Purpose/Objectives: Cancer treatment survivors often report impaired functioning and increased falls. Not all survivors experience the same symptom burden, suggesting individual susceptibilities. *APOE* genotype is a potential genetic risk factor for cancer treatment related side effects. Lifestyle factors such as physical activity can mitigate the effect of *APOE* genotype on measures of clinical interest in individuals without a history of cancer. We tested the hypothesis that *APOE* genotype influences cancer treatment related side effects and symptoms as well as response to exercise intervention.

Materials and Methods: Data from a subsample of a study of fall prevention exercise in post-treatment female cancer survivors aged 50–75 years old (https://clinicaltrials.gov NCT01635413) were used to conduct a secondary data analysis. ApoE genotype was determined by serum sampling. Physical functioning, frequency of falls, and symptom burden were assessed using survey instruments.

Results: Data from 126 female cancer survivors a median of 49 months out from cancer diagnosis were analyzed. ApoE4 carriers trended toward a higher fall rate at baseline (*p* = 0.059), but after exercise intervention had a fall rate lower than E4 non-carriers both immediately after structured intervention (*p* = 0.013) and after 6 months of follow up (*p* = 0.002). E2 carriers did not show improved measures of depressive symptoms and self-report disability after exercise intervention. E3 homozygotes showed increased self report physical activity after the 6 month exercise intervention, but E4 and E2 carriers did not.

Conclusions: *APOE* genotype may modulate cancer treatment related side effects and symptoms and response to exercise intervention.

## INTRODUCTION

The number of cancer survivors in the United States continues to increase due to a growing, aging population and from improved cancer detection and treatment. As of January 2019, there were an estimated 17 million cancer survivors living in the United States [1]. Within 10 years, the number of cancer survivors in the US is projected to increase by 31% [1], meaning more people will be living longer with the side effects of cancer and cancer treatment. Many of these cancer survivors will be elderly and may experience reduced quality of life from a preventable or treatable toxicity related to cancer or cancer treatment. Although these long-term effects are often attributed to cytotoxic chemotherapy, the underlying mechanisms are multifactorial and may also include contributions of the malignancy itself, surgery, radiotherapy, hormonal therapy, immunotherapy, and targeted therapy [2–4]. It is important to understand how individual genetic susceptibility factors influence symptom burden and efficacy of mitigation strategies in cancer survivors to best tailor rehabilitation programs.

Cancer survivors often experience impaired functioning even years after cancer treatment [5–7]. Frequently reported symptoms include behavioral and cognitive changes such as difficulty concentrating, memory impairment, fatigue, and increased anxiety [8, 9]. These symptoms are important not only to quality of life, but also have been associated with increased risk of falls, decreased activity levels, and increased disability [10–13]. Functional impairments and increased risk of fall have also been associated with chemotherapy-induced peripheral neuropathy [14]. Importantly, there may be subsets of survivors who are particularly vulnerable to late functional impairments as a result of cancer treatment [5, 15].

Assessing genetic factors of neurological vulnerability may increase the understanding of behavioral and cognitive impairments among cancer survivors. One such genetic risk factor is apolipoprotein E (apoE) isoform [16]. ApoE is involved in cholesterol and lipid homeostasis and synaptic functions [17]. More recently, the role of apoE in immunomodulation, especially in the central nervous system (CNS), has also been recognized [18]. There are three major isoforms of apoE present in humans: E2, E3, E4. In the general population, E3 is encoded by far the most commonly possessed ε3 allele (79.8%), followed by E4 that is encoded by the ε4 allele (14.9%) and E2 encoded by the least common ε2 allele (5.3%).[19]. Possession of one or two ε4 alleles is associated with increased risk of developing cardiovascular disease, Alzheimer’s disease (AD), and cognitive impairments following various environmental challenges [20–23]. Even among healthy middle-aged populations, compared to ε3, possession of an ε4 allele is associated with accelerated cognitive decline [24]. Compared to E3, E2 is associated with a relative protective effect in risk to develop AD, but is associated with increased propensity toward developing more severe symptoms in survivors with post-traumatic stress disorder (PTSD) [19]. Less is known regarding the role of apoE isoform in influencing cancer survivorship. Several studies have identified an association between the ε4 allele and increased vulnerability to cognitive dysfunction after cancer treatment in survivors with breast cancer, lymphoma, and testicular cancer receiving chemotherapy [16, 25–27]. as well as in survivors with brain tumors [28, 29].

Prior studies have identified the impact of lifestyle-related factors on mediating the relationship between apoE isoform and long-term cancer-related toxicity. Remarkably, smoking has been identified as a protective factor against cancer-related cognitive impairment among apoE4 carriers [25, 29]. However, despite recognition of exercise as a salient protective factor against functional decline in apoE4 carriers in the setting of other medical comorbidities, this relationship has not yet been explored in the context of cancer [30, 31]. Epidemiologic evidence suggests that exercise may not only curb side effects during active cancer treatment, but may also lower the risk of cancer recurrence and improve quality of life in cancer survivors [32–34]. Exercise has cardio-metabolic benefits [35] and may also attenuate the increased risk of cardiovascular disease following cancer treatment, which is now a competing cause of morbidity and mortality for female cancer survivors [36–38].

In the current analyses, the modulating effect of apoE genotype on functional status and symptom burden in response to exercise intervention was investigated in a subsample of trial participants. Data were analyzed from the GET FIT (“Group Exercise Training for Functional Improvement after Treatment;” NCT01635413) study, a single-blind, parallel group, prospective controlled trial involving randomized underactive female cancer survivors previously treated with chemotherapy assigned to one of three study arms: (1) tai chi training, (2) lower body strength training, or (3) an exercise placebo (stretching and relaxation classes) [39]. More specifically, we testes the hypothesis that apoE isoform modulates cancer- and cancer treatment-related side effects and symptoms in response to exercise intervention.

## RESULTS

### Participant characteristics

*APOE* genotyping was performed on 133 participants in the GET FIT trial. Seven genotyped individuals were excluded for having the E2/E4 genotype, due to conflicting literature on relative advantages of the E2 and E4. *APOE* allele frequency data are shown in the Supplementary Table 1. Seventeen participants carried an ε2 allele while 29 carried an ε4 allele. To determine whether there was an enrichment of any specific genotype in our participant pool, *APOE* allele frequencies were plotted against reference frequencies in the general population using chi-square goodness of fit. The study population demonstrated similar allele frequencies to a reference population [19] for E2 (6.7% vs. 5.3%) and E4 (11.5% versus 14.9%) [Chi-Square = 3.06, *p* = 0.217].

Participant characteristics are shown in Table 1. The median age at enrollment in the trial was 65 years (interquartile range [IQR] 61.4 to 68.7 years) with a median time since cancer diagnosis of 49 months (IQR 18.3 to 79.8 months). The cohort was largely non-Hispanic (97.5%, 121/124; 2 declined to answer), Caucasian (89.7%, 113/126), and highly-educated (49.2% with undergraduate or postgraduate degree, 62/126). Most participants were married (57.9%, 73/126) and many were retired (49.2%, 62/126) at the time of study enrollment. E4 carriers were younger (mean age at enrollment 61.1 vs. 64.2; *p* = 0.012) and had been diagnosed with cancer at an earlier age (mean age at diagnosis 55.6 vs. 59.0; *p* = 0.015) than non-E4 carriers. There were no differences in participant characteristics between E2 carriers and non-carriers.

**Table 1:** Demographic data of study participants (*n* = 126)

	Genotype			
	E2−	E2+	E4−	E4+
Age at enrollment *Median (IQR)*	64.0 (60–68)	66.0 (62.3–69.8)	66.0 (62.3–69.8)	62.0 (56.5–67.5)
Age at cancer diagnosis *Median (IQR)*	58.0 (52.4–63.6)	60.6 (58.9–62.4)	59.1 (54.3–63.9)	55.8 (49.8–61.9)
*Ethnicity*				
Hispanic	3% (3)	0% (0)	3% (3)	0% (0)
Non-Hispanic	96% (105)	94% (16)	96% (93)	97% (28)
Decline to answer	1% (1)	6% (1)	1% (1)	3% (1)
*Race*				
Caucasian/White	89% (97)	94% (16)	90% (87)	90% (26)
African-American/Black	1% (1)	6% (1)	1% (1)	3% (1)
Native Hawaiian/Pacific Islander	1% (1)	0% (0)	0% (0)	3% (1)
American Indian/Alaskan Native	2% (2)	0% (0)	1% (1)	3% (1)
Asian	2% (2)	0% (0)	2% (2)	0% (0)
More than 1 race	6% (6)	0% (0)	6% (6)	0% (0)
*Highest degree attained*				
High school diploma	24% (26)	35% (6)	23% (22)	34% (10)
Associate/technical	26% (28)	24% (4)	30% (29)	10% (3)
Undergraduate degree	28% (30)	24% (4)	25% (24)	34% (10)
Postgraduate degree	23% (25)	18% (3)	23% (22)	21% (6)
*Marital status*				
Married/Partnered	60% (65)	47% (8)	56% (54)	66% (19)
Divorced/Separated	19% (21)	29% (5)	21% (20)	21% (6)
Widowed	12% (13)	24% (4)	16% (16)	3% (1)
Single	9% (10)	0% (0)	7% (7)	10% (3)
*Employment*				
Retired	47% (51)	65% (11)	51% (49)	45% (13)
Full time	21% (23)	18% (3)	22% (21)	17% (5)
Part time	20% (22)	12% (2)	18% (17)	24% (7)
Homemaker	5% (5)	6% (1)	5% (5)	3% (1)
Unemployed	7% (8)	0% (0)	5% (5)	10% (3)
Total	100% (109)	100% (17)	100% (97)	100% (29)

Disease and treatment characteristics of the subsample are listed in Table 2. Breast cancer was the most common cancer diagnosis (69%, 87/126). Most participants had early stage cancer (61.9%, 78/126 with stage I or II disease) and received multimodal therapy including radiation (65.1%, 82/126) and/or surgery (88.1%, 111/126) in addition to chemotherapy (100%, 126/126).

**Table 2:** Participant cancer and cancer treatment history (*n* = 126)

	Genotype			
	E2−	E2+	E4−	E4+
Cancer type	***n* (%) **			
Breast	77 (71)	10 (59)	67 (69)	20 (69)
Cervical	3 (3)	0 (0)	1 (1)	2 (7)
Colon	6 (6)	2 (12)	6 (6)	2 (7)
Lung	3 (3)	2 (12)	5 (5)	0 (0)
Lymphoma	5 (5)	1 (6)	4 (4)	2 (7)
Ovarian	6 (6)	1 (6)	6 (6)	1 (3)
Uterine	5 (5)	1 (6)	4 (4)	2 (7)
Other	4 (4)	0 (0)	4 (4)	0 (0)
Cancer stage				
I	29 (27)	6 (35)	24 (25)	11 (38)
II	39 (36)	4 (24)	34 (35)	9 (31)
III	30 (28)	5 (29)	30 (31)	5 (17)
IV or metastatic	0 (0%)	0 (0)	0 (0%)	0 (0)
No stage/don’t remember	11 (10)	2 (12)	9 (9)	4 (14)
Cancer treatment received	108 (99)	16 (94)	96 (99)	28 (97)
Chemotherapy	109 (100)	17 (100)	97 (100)	29 (100)
Surgery	99 (91)	13 (76)	85 (88)	27 (93)
Radiation	74 (68)	9 (53)	63 (65)	20 (69)
Hormone therapy	40 (37)	3 (18)	33 (34)	10 (34)
Diagnosed with any other type of cancer?	21 (19)	3 (18)	21 (22)	3 (10)
Total	109 (100)	17 (100)	97 (100)	29 (100)

### Fall data

Thirty five participants recalled a fall in the 6 months prior to study enrollment. Of these 35 participants, 11 did not experience another fall, 10 fell again during the 6 month exercise intervention time period, 8 fell again during the 6 month post-intervention follow up time period, and 6 fell again during both the intervention and post-intervention time points. Of the 86 participants who reported no recent falls at the time of enrollment, 50 went on to report no future fall events. Of the remaining 36 participants who had not fallen recently prior to enrollment, 20 fell during the intervention time period only, 12 fell during the post-intervention time period, and 4 fell during both time periods.

There were no differences in fall rates seen between exercise groups. Therefore, these groups were collapsed for analysis of fall rate. In order to compare between the different genotype groups, a standardized fall rate per 1000 participant-days was calculated and is shown in Table 3 broken down by E2 and E4 carrier groups. In the 6 months prior to enrollment, or any intervention and based on participant recall, there was a trend toward a higher fall rate among E4 carriers than non-E4 carriers (2.52 per 1000 participant-days versus 5.56, *p* = 0.059). By the final month of the study (Month 12), there was a decrease in fall rate per 1000 participant-days among all participants (*p* = 0.043). However, this effect was significantly greater in E4 carriers than non-E4 carriers at both measurements during the 9-month (*p* = 0.013) and 12-month (*p* = 0.0002) post-intervention time points.

**Table 3:** Exercise intervention significantly decreases fall rate in E4 carriers

Fall rate per 1,000 participant-days^1^							
Genotype	Month	*N*	Mean fall rate	Genotype	Month	*N*	Mean fall rate
E2−	B	109	3.47	E4−	B	97	2.52
	1	109	4.89		1	97	5.50
	3	106	1.89		3	94	2.13
	6	105	2.54		6	93	2.87
	9	105	1.59		9	93	2.15
	12	105	2.54		12	93	3.94
E2+	B	17	1.63	E4+	B	29	5.56
	1	17	7.84		1	29	4.60
	3	16	4.17		3	28	2.38
	6	16	6.25		6	28	3.57
	9	16	2.08		9	28	0^*^
	12	16	6.25		12	28	0^***^

^1^Fall rate was calculated as (# participant-falls/# participant-days) × 1000. The fall rate of E4 carriers per 1000 participant-days decreased from 5.56 falls per 1000 participant days to 0 from baseline to month 12 (*p* = 0.043). There was a trend toward higher fall rate among E4 carriers versus E4 non-carriers at baseline (2.52 per 1000 participant-days versus 5.56, *p* = 0.059). However, after exercise intervention, E4 carriers demonstrated a significantly lower fall rate when compared with E4 non-carriers at both the 9 (2.15 versus 0, ^*^ *p* = 0.013) and 12 month (3.94 versus 0, ^***^ *p* = 0.002) time periods. Abbreviation: B: baseline.

### Cancer treatment related side effects and symptoms

**Figure 1:**
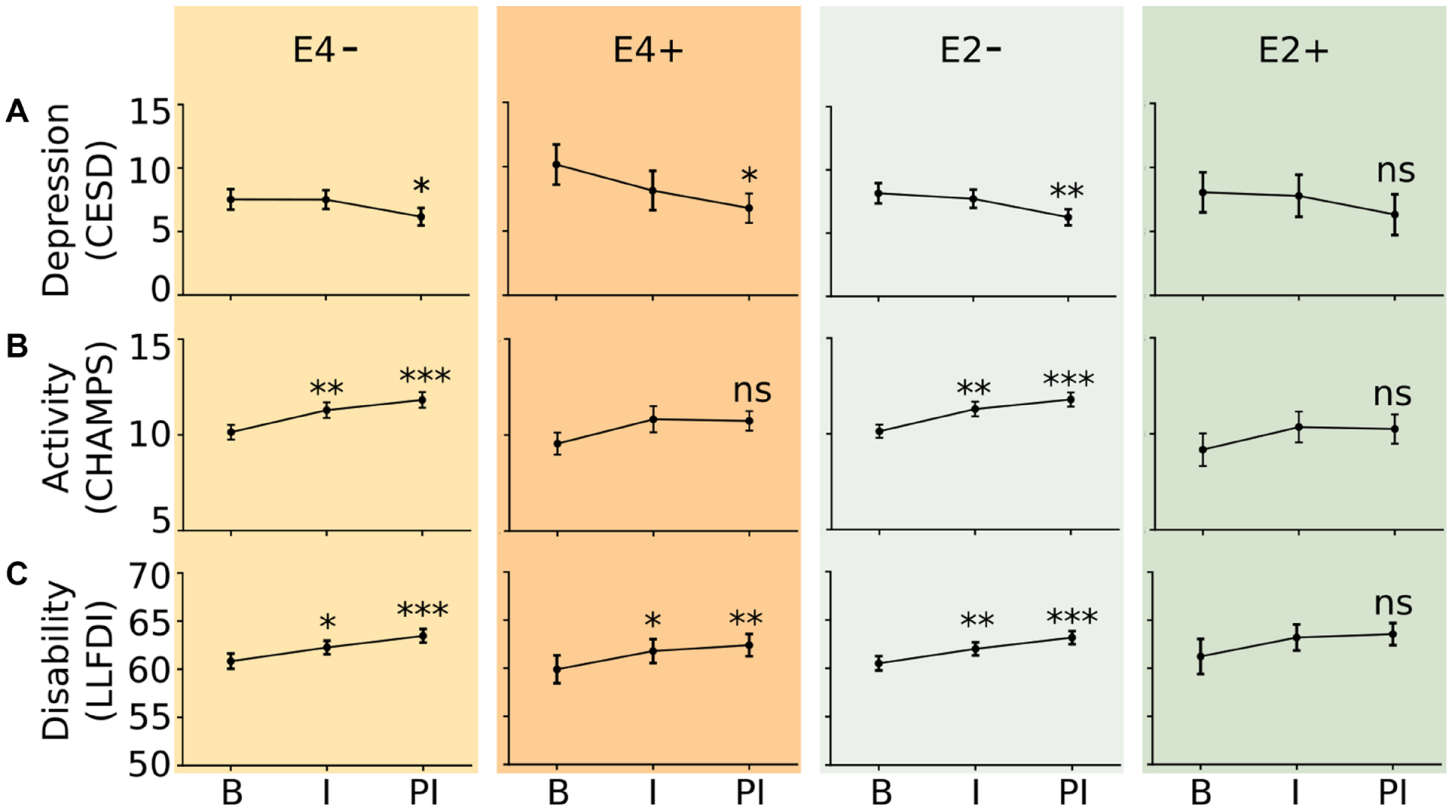
Effect of exercise intervention on survey indices of depressive symptoms (**A**), activity (**B**), and physical disability (**C**) during the baseline (B), intervention (I), and post-intervention (PI) timepoints. Depressive symptoms were measured by the Center for Epidemiological Studies Depression Scale. Activity was measured using the CHAMPS Activity Questionnaire for Older Adults; Physical disability was measured using the Late Life Functionality and Disability Instrument Limitation Questionnaire. E4−: *n* = 87 study participants; E4+: *n* = 27 study participants; E2−: *n* = 99 study participants; E2+: *n* = 16 study participants. ^*^ *p* < 0.05, ^**^ *p* < 0.05, ^***^ *p* < 0.005 versus B.

There were no differences in changes in self-reported depressive symptoms, physical activity, and physical functioning by exercise intervention arm. Therefore, these groups were collapsed for further analysis. Assessments were then compared by E4 and E2 carrier status (Figure 1). E4 carriers had a reduction in depressive symptoms over the entire 12 month study time period, as measured by the CESD (*p* = 0.003) but E2 carriers did not (*p* = 0.244). Non-E2 and non-E4 carriers demonstrated improved activity levels after the 6 month exercise intervention as measured by the CHAMPS questionnaire, while those who possessed at least one ε2 or ε4 allele did not demonstrate significant change. Except for E2 carriers, all genotype groups demonstrated significant improvement in the disability component LLFDI questionnaire, meaning that they could perform more activities independently than before.

### Neuropathy data

**Figure 2:**
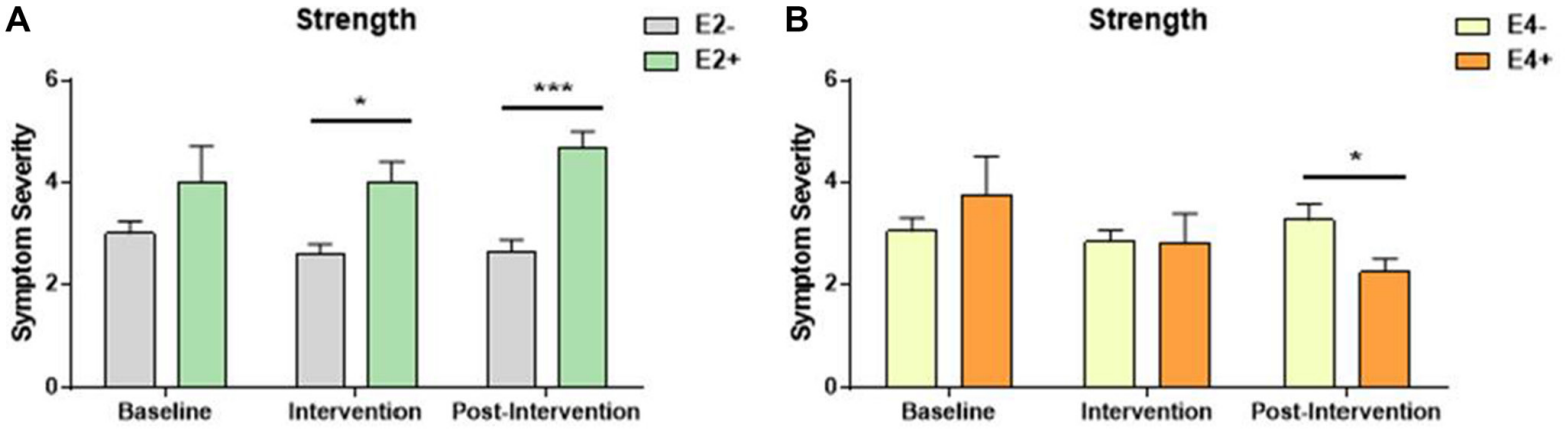
Symptom severity of individuals who reported neuropathy symptom incidence. Participants ranked severity between 2 and 5, with 5 being the most severe. (**A**) E2 carriers in the strength intervention experienced significantly more neuropathy symptoms than E2 non-carriers both during and after the intervention time point. ^*^ *p* = 0.012, ^***^ *p* = 0.003, respectively. (**B**) During the post-intervention timepoint, the opposite pattern was noted with E4 carriers experiencing less neuropathy symptoms in the strength intervention group. ^*^ *p* = 0.014).

Among participants who reported neuropathy symptoms, neuropathy symptom severity was similar at baseline among all intervention and genotype groups (Figure 2A and 2B, Table 4). However, among participants who underwent the strength training intervention, those who lacked E2 had significantly lower neuropathy severity than heterozygous or homozygous E2 carriers, both during the exercise intervention and during the post-intervention time periods (Figure 2A). In the stretching control group, E2 carriers reported lower neuropathy severity in the post-intervention time period compared to non-E2 carriers (3.56 versus 2.00, *p* < 0.001; Table 4). E4 carriers in the strength training group had significantly lower neuropathy severity scores during the post intervention time point than non-E4 carriers (Figure 2B).

**Table 4:** Neuropathy symptom burden noted by *APOE* genotype in the flexibility and Tai chi training group before, during, and after intervention

Exercise Group	Genotype	Baseline		Intervention		Post-intervention	
		Severity	*N*	Severity	*N*	Severity	*N*
Flexibility	E2−	3.53 ± 0.30	17	3.53 ± 0.30	17	3.56 ± 0.27	18
	E2+	2	1	3.00	2	2.00	3
	*p*	0.245		0.095		<0.001	
	E4−	3.43 ± 0.34	14	3.53 ± 0.29	15	3.38 ± 0.32	16
	E4+	3.50 ± 0.65	4	3.25 ± 0.75	4	3.20 ± 0.49	5
	*p*	0.926		0.743		0.772	
Tai chi training	E2−	3.18 ± 0.246	17	3.28 ± 0.30	18	3.06 ± 0.21	18
	E2+	4.50 ± 0.500	2	4.00 ± 1.0	2	2.50 ± 0.50	2
	*p*	0.177		0.599		0.452	
	E4−	3.46 ± 0.33	13	3.33 ± 0.32	15	3.07 ± 0.25	14
	E4+	3.00 ± 0.26	6	3.40 ± 0.60	5	2.83 ± 0.31	6
	*p*	0.289		0.925		0.556	

## DISCUSSION

This is the first examination of the relationship among exercise, *apoE* genotype, and side effects and symptoms of cancer in a subset of older female cancer survivors participating in a large clinical exercise trial. The study findings suggest that *APOE* genotype may be associated with presence and severity of cancer treatment-related side effects and symptoms and also influence the response to exercise-based interventions in cancer survivors.

Cancer survivors carrying at least one ε4 allele fell less after undergoing exercise intervention both in comparison to their baseline and to non-E4 carriers. The same pattern was not seen when comparing E2 and non-E2 carriers. Cancer survivors who carry an ε4 allele seem to experience greater benefit from a strength training program in terms of neuropathy symptom burden than those who do not carry an ε4 allele. However, the opposite trend is shown in E2 carriers: participants carrying an ε2 allele have significantly higher neuropathy symptom burden when compared to non-E2 carriers after a strength training intervention. This ε4 effect was not seen after tai chi training or flexibility control intervention.

*APOE* genotype may modulate long-term cancer-related toxicity through a number of pathways. Cancer treatment may cause CNS injury through vascular damage, depletion of glial progenitor cells, oxidative stress, neuroinflammation, demyelination, and disruption of hippocampal neurogenesis [40, 41]. The pleiotropic effects of apoE involving the hypothalamic-pituitary-adrenal axis [19, 42], and the immune system [43] may modulate these effects [43]. Previously, E3 has been described as functioning in an antitumor capacity through suppression of angiogenesis and cell invasion [44], while E2 has been associated with decreased risk of gastric cancer [44–46]. A recent study found E4 was associated with significantly prolonged survival in survivors with melanoma, while E2 was associated with shorter survival [43].

Our study does not show long-term differences after cancer treatment in symptoms of depression, functional status, or neuropathy symptoms burden based on E2 or E4 status. This finding aligns with several prior studies [47–52]. The dissociation between the effects of E4 status on a reduction in falls but not on depressive and neuropathy phenotypes suggests that this reduction in falls might be related to improved vestibular or motor function in E4 carriers following exercise. Consistent with this beneficial effect of exercise in E4 carriers, in patients with mild Alzheimer’s disease, E4 carriers benefitted more from physical exercise than non-E4 carriers with regard to improvement in physical and cognitive measures [53].

In female breast cancer survivors, those with a history of falls at baseline performed worse when integration of vestibular input was critical for maintaining balance, but balance problems at baseline did not predict falls over six month [54]. Similarly, in prostate cancer survivors a history of falls but not balance at baseline predicted falls over twelve month [55]. In contrast, in a study of female and male cancer survivors, impaired balance predicted falls over twenty four months [56]. In the context of those studies, it is remarkable that in the current study the beneficial effect of exercise on falls in E4 carriers is seen while there was a trend toward a higher fall rate in E4 carriers than E4 non-carriers at baseline.

The current study has the following limitations. The first limitation is the sample size of the subsample and the relatively low numbers of E2 or E4 carriers. It is possible that our study was not sufficiently powered to detect relatively subtle differences between E2 or E4 carriers versus non-carriers, but sufficiently large to reveal this difference once exercise intervention was introduced. We could not explore dose-effect relationships, as our study population did not contain any survivors with the E4/E4 or E2/E2 genotypes. Another limitation is that only women were included in the fall prevention exercise in post-treatment cancer survivors study. Future studies of the relationship of *APOE* genotype with long-term toxicity burden and functional outcomes in male cancer survivors enrolled in a clinical exercise trial are warranted.

## MATERIALS AND METHODS

### Study design

After approval by the Oregon Health & Science University (OHSU) institutional review board, 444 female participants were enrolled and provided informed consent between September 2012 and October 2016. After baseline testing women were randomly assigned to one of three exercise groups: strength training, tai chi training, or the stretching control group. An ancillary study was conducted to compare changes in inflammatory markers in response to each of the exercise interventions. Participation in the ancillary study included an additional blood draw and was completely voluntary. For 133 of these ancillary study participants serum samples, were available for *APOE* genotyping and were considered for the current analysis.

### Study population

Participant recruitment for the GET FIT trial has been previously described [39]. Inclusion criteria included the following: female sex, diagnosed with stage I-IIIc cancer other than cancers of the brain or spinal cord, completion of chemotherapy >3 months prior to enrollment, no ongoing adjuvant therapy other than hormone therapy for breast cancer, aged 50–75 on the date of enrollment, underactive at baseline (<60 minutes of moderate intensity exercise per week at the time of enrollment), cognitive ability sufficient to answer survey questions and to participate in exercise classes, and free of any medical condition that contraindicates participation in moderate intensity exercise. Exclusion criteria included male sex.

### Participant assessments

At the time of enrollment, participants self-reported their demographics and medical history using an in-house questionnaire. Participants were also asked to complete survey questionnaires including the Functional Comorbidity Index (FCI; a self-administered 18-item scale of comorbidities effect on physical functioning) [57], Late Life Function & Disability Instrument (LLFDI; an assessment of functional limitations and performance of socially defined life tasks) [58], the Community Healthy Activities Model Program for Seniors Activity Questionnaire for Older Adults (CHAMPS; an assessment of weekly frequency and duration of lifestyle physical activities) [59], and Center for Epidemiological Studies Depression Scale (CES-D; a screening test for depression and depressive disorder) [60]. Participants were also asked about current symptom burden including neuropathy. These assessments were repeated at the 3 month (mid-intervention), 6 month (post-intervention), and 12 month (6 month follow up to supervised training) data collection visits. Falls during the study period were assessed prospectively by monthly reports [39]. A fall was defined as unintentionally coming to rest on the ground, not as a result of extenuating circumstances. Baseline fall rate was assessed through a 6-month recall.

### Exercise interventions

The exercise protocols used in the GET FIT study have been previously published in detail [39]. The strength training program was based on training programs that improved neuromuscular function and reduced fall risk factors in our prior studies in women without cancer [61]. The tai chi training protocol consisted primarily of 8 purposeful movement forms, developed on the basis of the original simplified 24-form Yang-style tai chi training and also shown to prevent falls in non-cancer populations [62, 63]. In the exercise placebo stretching group, participants performed a series of seated or lying whole body flexibility and progressive neuromuscular relaxation exercises of the same frequency, duration, and length as the other groups, but intended to have little effect on fall risk factors [64]. Participants in each study group attended supervised one hour classes two days per week for six months, and were followed for an additional six months after the supervised intervention period finished.

### Genotyping

Serum samples collected from the study participants were used for *APOE* genotyping, as previously described [65]. *APOE* genotypes were determined by Dr. Clive Woffendin at the Oregon Clinical Translational Research Institute (OCTRI) of OHSU using the Oragene self-collection methodology (DNA Genotek Inc., Ottawa, ON, Canada) as previously described [66]. Following polymerase chain reaction (PCR) amplification and restriction digestion with HhaI, DNA fragments were resolved on an 8% polyacrylamide nondenaturing gel, stained with ethidium bromide and visualized by ultraviolet illumination. Sizes of HhaI fragments were estimated by comparison with DNA size markers and the *APOE* genotype determined according to the unique pattern for each isoform. Known control samples of each *APOE* genotype were run alongside the unknown samples in each genotyping procedure.

### Statistical analysis

Participants were divided into carriers versus non-carriers for E2 (E2+ versus E2−) and E4 (E4+ versus E4−). Individuals with the E3/E3 genotype were included as non-carriers in both analyses. Continuous data between groups were compared with a two-sample *t* test. Fisher’s exact test was used to compare categorical variables. Repeated measures ANOVA were used to evaluate continuous variables over the study time period. Percentages were rounded to the nearest percentage point. Means ± SEM are reported. All tests were two-sided, and *p* values of <0.05 were considered significant. SPSS Statistical Software v25 (Chicago, IL, USA) was used for statistical analysis and Graphpad Prism software (San Diego, CA, USA) for the generation of the figures.

## CONCLUSIONS

Long-term cancer survivorship is increasingly common. Cancer survivors often lose functional ability and experience behavioral and cognitive dysfunction. The aim of the present investigation was to examine the relationship of E2 and E4 with long-term toxicity burden and functional outcomes in a sample of female cancer survivors treated with chemotherapy enrolled in a clinical exercise trial. Our data suggest that *APOE* genotype determines who may benefit the most from exercise interventions in long-term measures of mood, functional status, and toxicity burden. E4 carriers appear to benefit significantly from a strength training intervention. Increased efforts are warranted to assess the role of apoE isoforms in cancer survivors and the mechanisms underlying these apoE isoform-dependent effects.

## SUPPLEMENTARY MATERIALS


