# Supplementary material for: Association of fall rate and functional status by APOE genotype in cancer survivors after exercise intervention
Source: Oncotarget. 2022 Nov 17;13:1259–70. doi: 10.18632/oncotarget.28310 (PMC11623406; doi:10.18632/oncotarget.28310)
Supplement: Supplementary file 1 [file oncotarget-13-28310-s001.pdf]

Association of fall rate and functional status by *APOE* genotype in cancer survivors after exercise intervention

SUPPLEMENTARY MATERIALS

Supplementary Table 1: *APOE* allele frequencies compared to reference[1]

APOE Allele	Frequency (*n*)	Reference frequency	*p*
ε2	6.7% (17)	5.3%	0.477
ε3	81.7% (206)	79.8%	
ε4	11.5% (29)	14.9%	

[1]Data from: Johnson LA, Zuloaga DG, Bidiman E, Marzulla T, Weber S, Wahbeh H, Raber J. apoE2 exaggerates PTSD-related behavioral, cognitive, and neuroendocrine alterations. Neuropsychopharmacology. 2015; 40:2443–53.
